# Dynamic Chemistry Toolbox for Advanced Sustainable Materials

**DOI:** 10.1002/advs.202308666

**Published:** 2024-02-06

**Authors:** Yuanxin Deng, Qi Zhang, Ben L. Feringa

**Affiliations:** ^1^ Key Laboratory for Advanced Materials and Feringa Nobel Prize Scientist Joint Research Center School of Chemistry and Technology 130 Meilong Road Shanghai 200237 China; ^2^ Stratingh Institute for Chemistry and Zernike Institute for Advanced Materials Faculty of Science and Engineering University of Groningen Nijenborgh 4 Groningen 9747 AG The Netherlands

**Keywords:** chemically recyclable polymers, dynamic chemistry, green plastics, sustainable materials

## Abstract

Developing dynamic chemistry for polymeric materials offers chemical solutions to solve key problems associated with current plastics. Mechanical performance and dynamic function are equally important in material design because the former determines the application scope and the latter enables chemical recycling and hence sustainability. However, it is a long‐term challenge to balance the subtle trade‐off between mechanical robustness and dynamic properties in a single material. The rise of dynamic chemistry, including supramolecular and dynamic covalent chemistry, provides many opportunities and versatile molecular tools for designing constitutionally dynamic materials that can adapt, repair, and recycle. Facing the growing social need for developing advanced sustainable materials without compromising properties, recent progress showing how the toolbox of dynamic chemistry can be explored to enable high‐performance sustainable materials by molecular engineering strategies is discussed here. The state of the art and recent milestones are summarized and discussed, followed by an outlook toward future opportunities and challenges present in this field.

## Graphical abstract

Plastics bring problems: Is dynamic chemistry a solution?In this review, a collection of emerging molecular tools including noncovalent and dynamic covalent bonds and building blocks for the development of sustainable materials with advanced performance are summarized.

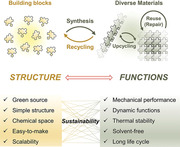

## 1 Introduction

Plastics have been considered to be among the most successfully designed materials transforming society since their invention in the middle of the 19th century. However, the widespread application and frequent abuse of traditional plastics made from fossil sources has caused a series of problems in energy, environment, and public health due to the difficulties in degradation and reuse.^[^ ^1^ ^]^ This fact causes an urgent social need for the replacement of traditional plastics by developing a more sustainable and environmentally friendly plastic economy. To achieve this goal, several strategies and solutions have been proposed and research conducted both in academia and industries generally focusing on three aspects: i) developing biobased polymer materials to replace the use of fossil sources;^[^ ^2^ ^]^ ii) endowing materials with durability and reusability to elongate the life cycle;^[^ ^3^ ^]^ iii) achieving economically efficient and waste‐free procedures for chemical recycling and upcycling of polymeric materials.^[^ ^4^, ^5^ ^]^ Milestones have been achieved in using green catalysis to enable these goals, such as catalytic refining of traditional plastics into value‐added products, reprocessing a cross–linked network (vitrimer) by catalytic activation of covalent bonds, and catalyst‐enabled chemical recycling of traditional plastics.^[^ ^6^, ^7^, ^8^, ^9^, ^10^ ^]^ However, it remains highly challenging to design materials with the intrinsic ability to repair, reuse, and recycle,^[^ ^11^ ^]^ which requires constitutionally dynamic chemical bonds as the key structural elements in materials as well as effective delivery and amplification of dynamic functions from the microscopic to the macroscopic level. The key to this goal is to establish a versatile chemical toolbox that allows new dynamic functions integrated into the polymer design and fabrication without compromising material properties and robustness crucial to their wide range of applications.

**Figure 1:**
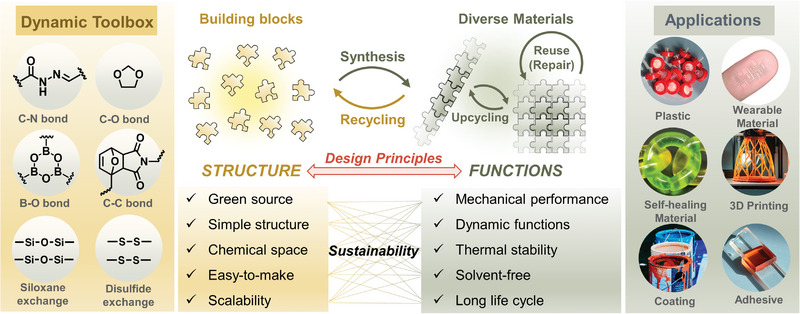
Conceptual illustration of dynamic chemistry enabling advanced sustainable materials.

Dynamic chemistry,^[^ ^12^ ^]^ including noncovalent and dynamic covalent chemistry,^[^ ^13^, ^14^, ^15^ ^]^ has rapidly developed and established a central position in chemistry and materials science in the past decades. It offers a chemical toolbox exploring the wealth of chemical bonding linkages featuring reversible dissociation/reformation, which is also the key to the complexity and dynamic functions of living systems.^[^ ^16^ ^]^ Bringing this concept to the design of sustainable polymers, one can expect that, by engineering the dynamic chemical bonds in the bulk materials, long polymers can readily reverse back into monomers by breaking the dynamic linkages that connect neighboring monomers within a polymer network which can also re‐organize cross–linking topologies to repair scratches and cracks on the materials (**Figure** **1**). These dynamic functions are exactly how nature achieves recycling sustainability in life systems using minimum amounts of feedstocks and reduced disposal (i.e., waste material).^[^ ^17^ ^]^ Nevertheless, despite the large family of dynamic chemistry tools established in solution,^[^ ^18^, ^19^ ^]^ only a small part of them have been introduced, in recent years, into the realm of sustainable polymers and material design.^[^ ^20^, ^21^, ^22^ ^]^ Meanwhile, the fundamental challenge rests on the inherent trade‐off between material performance and the dynamic nature of reversible chemical bonds, in which the introduction of relatively weak and dynamic bonds usually endows the materials with dynamic functions at the cost of loss of mechanical robustness and stability.^[^ ^23^, ^24^, ^25^ ^]^ This subtle trade‐off has been particularly evident and demanding facing the balance of dynamicity and material property, therefore often limiting the further application of dynamic materials after proof of concept.

In this context, our aims are to summarize recent progress and show opportunities in developing a dynamic chemical toolbox for the design of sustainable polymers and materials. This review starts with discussing several representative examples of dynamic chemistry principles (noncovalent and dynamic covalent bonds) that have been exploited and applied to advanced materials design in recent years. A few emerging strategies and design principles such as cooperating noncovalent and dynamic covalent chemistries in a single system will also be highlighted. Subsequently, state‐of‐the‐art representative examples will be discussed showing key aspects of dynamic functions, e.g., self‐healing, reprocessing ability, and chemical recyclability. Design principles will be discussed and attempts to overcome the challenge of how to construct robust and dynamic materials to increase the sustainability of materials. Finally, an outlook and perspective are given to identify future opportunities and challenges in this field. It should be emphasized that due to the limited space, only a selection of illustrative key examples from literature can be included in this review.

## 2 Emerging Dynamic Chemical Tools

The chemical basis of the dynamic chemical toolbox consists of a series of reversibly exchangeable noncovalent or covalent bonds. In this section, a family of dynamic chemical tools that emerged in the past decades will be summarized and presented from this perspective of designing sustainable polymers and materials. They will be divided by the bond types, intrinsic/extrinsic dynamicity (i.e., whether requiring the activation of external catalysts), the condensed phase of the materials (gels or solvent‐free polymers), and the specific condition for activating the dynamic bonds (e.g., temperature, pH, light, etc.). Besides the structural features, these factors and parameters are also essential for material design facing diverse properties and application scenarios. It should be noted that we don't attempt here to give a comprehensive review of all the chemical tools for dynamic materials, but aim at providing an overview of the state of the art of this molecular library to facilitate readers to search for the tools of interest.

### 2.1 Noncovalent Bonds

Noncovalent bonds widely exist in nature and are responsible for the complicated structures and functions of many bio‐macromolecules like proteins and DNA.^[^ ^16^ ^]^ The versatility and diversity of noncovalent chemistry have inspired generations of chemists to fabricate supramolecular assembled architectures that covalent synthesis cannot achieve. The concept of supramolecular chemistry pioneered by Lehn, Cram, and Pedersen opened a new era of dynamic chemistry based on noncovalent bonds.^[^ ^26^ ^]^ Ever since, a large family of noncovalent systems has been explored and introduced as reversible cross–linkers for polymers, including hydrogen bond,^[^ ^27^, ^28^, ^29^, ^30^ ^]^ metal‐ligand coordination,^[^ ^31^, ^32^ ^]^ host‐guest chemistry,^[^ ^33^, ^34^ ^]^ π‐π stacking,^[^ ^35^ ^]^ and ionic interactions^[^ ^36^ ^]^ (**Table** **1.1**). Some of them, e.g., host‐guest combinations, require solvents to solubilize or support the dynamic nature of noncovalent bonds, which might cause environmental impact due to the tedious purification and energy cost.^[^ ^37^ ^]^ Compared to pure organic solvent, water is more environmentally favorable and is frequently the solvent of choice for bio‐compatible materials.^[^ ^38^ ^]^ Incorporating water into a polymeric network can generate swellable soft materials, i.e., hydrogels, which exhibit many promising applications in biomaterials because of their composition and mechanical modulus akin to body tissues.^[^ ^39^, ^40^, ^41^, ^42^ ^]^ Besides soft matter, some recent examples also showed that taking advantage of multi‐length alignment and organization, some hydrogels featuring H‐bonds can be remarkably tough and strong, pushing further applications beyond hydrogels toward elastomers and even engineering materials.^[^ ^43^, ^44^, ^45^, ^46^ ^]^

**Table 1.1:** Representative examples of noncovalent (supramolecular) interactive bonds.

Interaction type	Molecular structure	Condition	References
H‐bonding	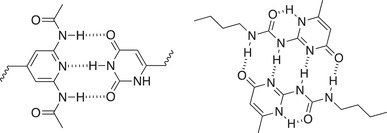	Room temperature (RT)	[27, 28, 29, 30]
Metal‐ligand coordination	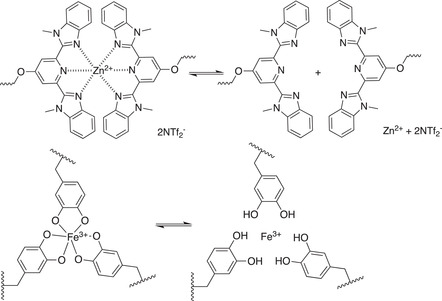	Ultraviolet light (UV) Force	[31, 32]
Host‐guest chemistry	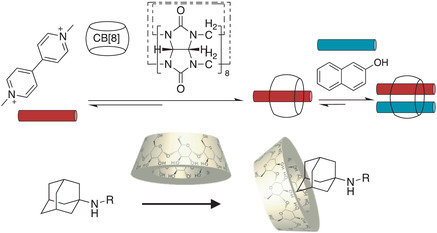	Water	[33, 34]
π–π stacking	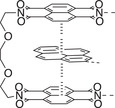	RT	[35]
Ionic interaction	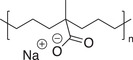	RT	[36]

H‐bonds are among the most investigated noncovalent bonds because of their omnipresence in biological systems and synthetic materials containing polar groups (e.g., amides).^[^ ^47^ ^]^ H‐bonds can be readily introduced using structurally simple moieties and they are easily engineered by tuning the number, steric hindrance, and geometry of H‐bond donors and acceptors. Many famous high‐performance industrial materials (e.g., Nylons and Kevlar) are made of polyamides featuring ordered assemblies of H‐bonds. Synthetic H‐bonding motifs and their applications in supramolecular polymeric materials have been pioneered by Lehn in the 90′s by using the triple H‐bonding interactions between DAD and ADA units (D refers to H‐bond donors; A refers to H‐bond acceptors).^[^ ^27^ ^]^ A superb H‐bonding motif invented by Meijer et al. is the 2‐ureido‐4‐pyrimidone (Upy) unit,^[^ ^29^ ^]^ featuring DDAA‐type self‐complementary quadrupolar H‐bonds, which has been extensively used in the construction of supramolecular polymers and materials.^[^ ^48^, ^49^, ^50^, ^51^ ^]^ The versatility and general interests of Upy units in emerging materials come from several distinctive features: i) their high binding constants up to 10^7^ M^−1^; ii) the preserved dynamic properties of Upy motifs in the solvent‐free network, i.e., not relying on solvated environments; iii) relatively simple structures easy to make (accessible building blocks at low‐cost).

Metal‐ligand interactions represent another useful noncovalent tool for designing dynamic materials.^[^ ^52^ ^]^ The high binding constant of metal‐ligand complexes usually stiffens the network by increasing the noncovalent cross–linking density. A few examples have demonstrated the effective enhancement of mechanical performances by introducing metal‐ligand cross–linkers (e.g., those based on iron(III), calcium, and zinc).^[^ ^53^, ^54^, ^55^ ^]^ It has been shown that these metal‐ligand complexes sometimes work unexpectedly well by simultaneously toughening and strengthening material properties in solvent‐free networks.^[^ ^56^, ^57^ ^]^ This toughening mechanism could be attributed to the mechanical dissipation mechanism of the dissociable metal‐ligand cross–linkers, also denoted as “sacrificial bonds” which break first to prevent the covalent fracture of polymer chains, thus enhancing the stretchability and toughness of the materials.^[^ ^58^ ^]^

Macrocyclic host‐guest chemistry also offers a class of noncovalent tools with unique structures and tunable binding constants.^[^ ^59^ ^]^ The key feature of the host‐guest combination is that it provides highly selective interactions,^[^ ^60^ ^]^ due to the specific host environment of the macrocycles, reminiscent of the highly selective molecular recognition between natural enzymes and their substances. Taking advance of these distinctive and reliable noncovalent interactions, self‐healing materials can be readily constructed.^[^ ^61^, ^62^ ^]^ Among them, water‐based host‐guest chemistry is especially attractive since it makes hydrogel materials frequently comprising water and minimal amounts of other molecules.^[^ ^63^, ^64^ ^]^ Recent progress by Scherman et al. showed how cucurbit[8]uril, a macrocycle that binds two guest molecules simultaneously, can act as a very robust supramolecular cross–linker for constructing high‐performance hydrogel materials.^[^ ^44^ ^]^

The interaction of π−π stacking has been long‐term used by supramolecular chemists to design one‐dimensional noncovalent assemblies, i.e., supramolecular polymers, in the solution phase.^[^ ^65^ ^]^ The weak nature of π−π interactions make them rarely used as a sole cross–linker for the design of supramolecular materials but usually act as a synergistic interaction with other noncovalent bonds, (e.g., metal‐coordination complexes^[^ ^66^ ^]^ and/or H‐bonds^[^ ^35^ ^]^). One of the most well‐known examples featuring such a synergy effect is the Kevlar material, which features an aromatic backbone linked with amide groups and thus exhibits excellent mechanical performance due to the synergistic cross–linking of amide H‐bonds and π−π stacking of aromatic backbone.^[^ ^67^, ^68^ ^]^

Ionic bonds widely exist in polyelectrolytes and other ionic polymers. The nature of ionic bonds is a strong interaction related to the Coulomb force of opposite charges that are spatially close to each other. Thus, materials fully cross–linked by ionic bonds are usually very robust. However, a large amount of soft matter, especially ionic gels, is reported and most of them exhibit soft and stretchable properties despite the strong cross–linking of ionic bonds.^[^ ^69^, ^70^, ^71^ ^]^ The key issue of ionic bonds in cross–linking materials might be their lability under the solvation conditions. As the ionic complexes, once formed, are usually very ordered (i.e., entropy disfavoured), the ionic bonds in a polymeric network are very sensitive to solvents. For example, water absorbed from the air can be such a solvent that plasticizes materials by solvating charged groups and inhibiting strong ionic bond formation, which, in many cases, actually endows materials with dynamic properties such as self‐healing and stretchability, but taking advantage of the solvent‐charge interactions instead of the ionic bonds.^[^ ^72^, ^73^ ^]^

Using supramolecular interactions to link small molecules can also result in high‐molecular‐weight macromolecular chains, also denoted as supramolecular polymers.^[^ ^30^, ^74^ ^]^ One of the key features of supramolecular polymers is their intrinsic recyclability,^[^ ^20^ ^]^ that is the reversibility to depolymerize into monomers under specific conditions (e.g., heating). However, although these concepts have been proven, the fact is that the disassembly or depolymerization, if happening at ambient temperatures, has inherent disadvantages, especially leading to instability of materials, which is observed in many cases of supramolecular polymers especially in the solution phase.^[^ ^30^ ^]^ To expand the applicable window of supramolecular polymers, noncovalent interactions with high binding constants are required as the main enthalpic force to form long polymers since the polymerization process is, in general, entropically disfavoured. However, enhancing the binding constants also leads to the unavoidable compromise on dynamicity, i.e., slower exchange kinetics. This situation results in a very subtle balance in which the thermodynamics and kinetic parameters of the global supramolecular systems should well fit the conditions (e.g., temperature, solvents) in the context of specific material applications.

A more straightforward strategy to enable sustainability by noncovalent bonds is to use them as reversible cross–linkers for covalent polymers.^[^ ^24^ ^]^ This design principle has been proven generally reliable in a wide range of materials such as gels, elastomers, adhesives, and other soft matter,^[^ ^75^, ^76^, ^77^ ^]^ which readily endows materials with self‐healing or reprocessability, but does not necessarily allow chemical recycling. Meanwhile, a noncovalent cross–linked network usually exhibits flowing behavior upon heating and lacks anti‐creeping ability. Notable progress (on processible and recyclable mechanically interlocked networks) has been made by Yan and coworkers by combining noncovalent interlocked molecules with covalent polymers to design high‐performance elastomers,^[^ ^78^, ^79^ ^]^ endowing supramolecular polymeric materials with excellent mechanical properties and added value.

### 2.2 Dynamic Covalent Bonds

Dynamic covalent bonds refer to a class of covalent bonds capable of dissociating and reforming reversibly under relatively mild conditions.^[^ ^15^, ^80^, ^81^ ^]^ Compared with conventional covalent bonds with high thermal stability, dynamic covalent bonds exhibit relatively low activation energy to exchange functional groups, while preserving the robust nature of covalent bonds, thus providing a type of dynamic chemical tools that stand at the intersection between noncovalent bonds and traditional covalent bonds.^[^ ^82^ ^]^ This feature, to some degree, overcomes the inherent trade‐off between dynamicity and robustness of dynamic materials. However, it should be noted that many dynamic covalent bonds (e.g., amide and ester bonds) are not intrinsically reversible like noncovalent bonds as the external addition of catalysts is required in many cases.^[^ ^83^ ^]^ This apparent drawback also offers opportunities to externally control the dynamic properties of originally robust covalent bonds in materials, which will be demonstrated with a few examples (vide infra in Section 3.2).

Some types of dynamic covalent bonds have been particularly investigated. The most well‐known and widely used example arguably is the dynamic imine bond^[^ ^84^ ^]^ which is formed by the condensation of an amide and an aldehyde. Various applications of dynamic imine chemistry were pioneered by Lehn, Stoddart et al.^[^ ^85^, ^86^ ^]^ in designing constitutional dynamic materials and discrete (supra)molecular assemblies and architectures, taking advantage of the highly efficient reaction and the self‐repairing and “error‐correcting” features during reversible exchange among the subcomponents. Recent advances towards enabling material sustainability (e.g., self‐healing ability, chemical recycling, reprocessing) by dynamic imine chemistry have been shown and several successful material applications have been reported.^[^ ^87^, ^88^, ^89^ ^]^

Furthermore, many other classes of dynamic covalent bonds have been explored in the past decades, including the S─S bond, Se─Se bond, C─N bond, C─O bond, B─O bond, C─C bond, and C─S bond (**Table** **1.2**).^[^ ^88^, ^90^, ^91^, ^92^, ^93^, ^94^, ^95^, ^96^, ^97^, ^98^, ^99^, ^100^, ^101^, ^102^, ^103^, ^104^, ^105^, ^106^, ^114^, ^115^, ^116^, ^117^, ^118^ ^]^ Specifically, these dynamic covalent reactions have been used for constructing healable or malleable materials taking advantage of the corresponding reversible bond formations, such as disulfide exchange, acylhydrazone exchange, imine transamination, and vinylogous urethane transamination, transesterification, boronic ester hydrolysis, and cycloaddition reactions.^[^ ^88^, ^90^, ^91^, ^94^, ^96^, ^102^, ^106^ ^]^ Many of them also offer reliable dynamic chemical tools for constructing self‐assembled architectures such as covalent organic frameworks, molecular cages, and knots.^[^ ^107^, ^108^, ^109^, ^110^, ^111^ ^]^ The intrinsic dynamic nature of some of these bonds also often enables the stimuli‐responsive capability of the resulting materials. For example, polymeric materials linked by disulfide bonds could be softened by irradiation of UV light to facilitate the reshaping or repairing under optical control.^[^ ^112^, ^113^ ^]^ The dynamic covalent bonds that involve the interference of water molecules usually respond to humidity and acidity effecting hydrolysis.^[^ ^86^ ^]^ Therefore, the dynamic functions that are presented by macroscopic materials with these functionalities incorporated could originate from the reversible exchange involving dynamic bonds and/or from the environmental conditions that trigger the bond exchange/dissociation reactions.

**Table 1.2:** Representative examples of dynamic covalent chemistry.

Reaction type	Chemical reaction	Condition	References
Dynamic exchange reaction	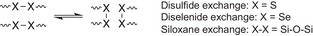	Red./oxid. Visible light Acid/base	[90, 99, 100]
Dynamic C‐N Bond			
Schiff‐base bond regeneration Acylhydrazone formation Oxime ester formation Aminal formation Amide exchange Imine transamination Vinylogous urethane transmamination	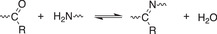 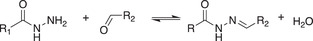 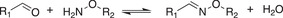 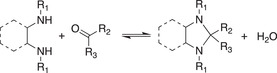 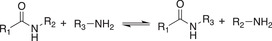 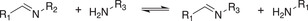 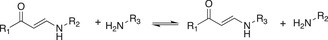	RT Heating/water/pH Excess alkoylamine/RT Water Catalyst Heat/catalyst Heat/catalyst	[101, 102, 103, 104, 105, 106]
Dynamic C‐O bond			
Transesterification Transcarbonation Cationoc ring opening polymerization of cyclic acetal	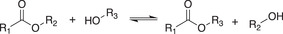 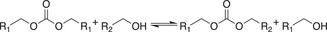 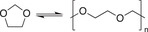	Heat/catalyst Catalyst Catalyst/strong H*	[91, 92, 93]
Dynamic B‐O bond			
Boronic ester hydrolysis	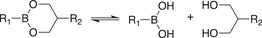	Water	[94]
Dynamic C‐C bond			
Diels‐Alder cycloaddition [2+2] cycloaddition reactions	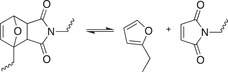 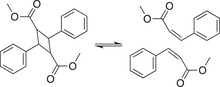	Heating hv	[95, 96]
Dynamic C‐S bond			
Thia‐Michael Thiourethane exchange	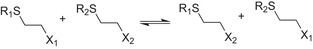 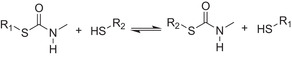	Heating/base Heating	[114, 115, 116, 117, 118]

A class of dynamic covalent chemistry based on C─S bonds has emerged in recent years. The representative examples include thia‐Michael (TM) additions and thiourethane exchange. Thia‐Michael additions have been well employed in the synthesis and postfunctionalization of polymers and their materials,^[^ ^114^ ^]^ for example, acting as the dynamic cross‐linker to design repairable and malleable polymeric materials.^[^ ^115^ ^]^ The resulting networks are inherently stable at room temperature while the dynamicity can be activated with catalytic amounts of bases or elevated temperature for bond exchange. To achieve a room‐temperature dynamic thia‐Michael exchange, Rowan and coworkers developed a series of isoxazolone‐based TM acceptors by taking advantage of aromatization upon thiol addition.^[^ ^116^ ^]^ On the other hand, thiourethane exchange is another inherently dynamic covalent bond. Usually, it is stable under ambient conditions, healable at moderate temperatures, and reprocessable at high temperatures. Thiourethane exchange was employed to design and synthesize a recyclable and solvolysable polythiourethane network by the facile catalyzed reaction of thiols and isocyanates.^[^ ^117^, ^118^ ^]^

Instead of using dynamic covalent bonds as reversible cross–linkers of polymers, another distinctive strategy to design intrinsically configurable polymers involves the combination of dynamic covalent chemistry with ring‐opening polymerization (ROP)^[^ ^119^, ^120^, ^121^, ^122^, ^123^, ^124^, ^125^ ^]^ to generate ROP dynamers. One of the representative examples is the disulfide‐mediated reversible ROP of cyclic disulfides. We reported a green chemical synthesis of poly(disulfide)s by solvent‐free one‐pot ROP of thioctic acid (TA)/lipoic acid (LA),^[^ ^119^ ^]^ a natural small molecule of low cost. The resulting materials exhibit excellent stretchability and self‐healing ability, due to the synergy of noncovalent bonds and dynamic covalent disulfide bonds in the solvent‐free network. Moreover, the five‐membered ring cyclic disulfides can be polymerized under neat conditions, while the depolymerization process can proceed by catalysis with base in aqueous solution.^[^ ^122^ ^]^ Virgin‐quality TA/LA monomers can be recovered by simple precipitation after acidification and filtration. By engineering the side chain, a series of polymers with diverse functions were prepared which show unique dynamic properties.^[^ ^120^, ^121^, ^123^, ^126^, ^127^ ^]^ A review of this emerging dynamic covalent chemistry has been reported,^[^ ^125^ ^]^ and a few very recent examples will also be discussed in Section 3.3. Meanwhile, a little progress has been made by developing catalyst‐enabled deconstruction of poly(acrylates) and reversible ring‐opening metathesis polymerization using Grubbs’ catalysts. These efforts offer another important strategy to achieve sustainable materials by catalytic design.^[^ ^128^, ^129^, ^130^, ^131^, ^132^, ^133^ ^]^

## 3 Dynamic Functions Enabling Sustainability

Functions, the key to adding value and enabling applications of materials, are central to material design especially for polymers because of their direct relationship between molecular structures and material properties. Making materials sustainable has become an important goal adding an aspect distinctive from all the conventional features, but also highly related to common functions. For example, robust mechanical properties result in high durability, which facilitates a longer life cycle of the applied materials (less disposal). The self‐healing ability allows scratched or cracked materials to repair automatically instead of becoming waste. Recyclability, including mechanical and chemical recycling, enables the direct use of plastic waste as feedstocks to produce monomers. All these properties facilitate the development of polymers that can be reprocessed readily, which is exactly how dynamic chemical tools can be used to accelerate the development of sustainable materials. This section will discuss very recent examples illustrating the state‐of‐the‐art in the field of advanced materials based on dynamic chemistry.

### 3.1 Self‐Healing Polymers

Self‐healing is an autonomous repairing process after physical or chemical damage. The reversible interactions needed for repairing could be based on non‐covalent or dynamic covalent bonds. Physical damage involves cleavage and slippage of the polymer chains. Segmental rearrangements and conformational changes or diffusion should then bring reactive chain ends into contact allowing them to repair the material.^[^ ^134^ ^]^ However, self‐healable materials are usually soft resulting in a limited application scope.^[^ ^135^ ^]^ A major challenge remains on how to design autonomously self‐healable materials with enhanced mechanical performances.^[^ ^136^, ^137^ ^]^ Thus, combining the features of mechanical robustness and self‐healing ability is a crucial goal when designing new polymeric networks to extend the practicality of such dynamic materials.^[^ ^137^, ^138^, ^139^ ^]^

**Figure 2:**
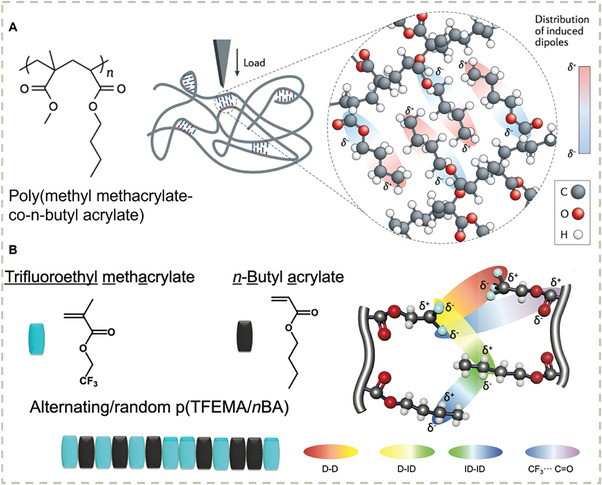
A) van der Waals interactions‐driven self‐healing copolymer, poly(methyl methacrylate‐*co*‐butyl acrylate). Reproduced with permission.^[^ ^140^ ^]^ Copyright 2018, The American Association for the Advancement of Science. B) Chemical structure of 2,2,2‐trifluoroethyl methacrylate (TFEMA) and n‐butyl acrylate (*n*BA) as well as the scheme of dipolar forces‐driven self‐healing poly(TFEMA/*n*BA) copolymers. Reproduced with permission.^[^ ^141^ ^]^ Copyright 2021, Wiley‐VCH GmbH.

The most ubiquitous supramolecular interaction is the van der Waals force, and it is inherent as non‐covalent cross–linkers in many high‐performance materials (e.g., high‐density polyethylene). Urban and coworkers designed new self‐healing copolymers based on the “key‐lock” cross–linkers using van der Waals forces.^[^ ^140^, ^141^ ^]^ Two readily available and commercially relevant monomers, methyl methacrylate (M) and butylacrylate (B), formed alternating copolymers that could efficiently self‐repair after mechanical damage without intervention (**Figure** **2**A). Interestingly, only copolymers with near‐equal incorporation of M and B showed efficient and autonomous healing. This phenomenon was attributed to the “key‐and‐lock” van der Waals interactions of interdigitating alkyl pendant groups between polymer chains. Modeling further suggested that the existence of helix‐like chain conformations and the interchain van der Waals forces triggered the self‐healing of poly(methyl methacrylate‐*co*‐butyl acrylate) materials. Although these dipolar forces are relatively weak compared to other noncovalent interactions, the ubiquitous intermolecular and/or intramolecular interactions running through the whole polymer network play a crucial role in achieving the mechanical integrity of polymeric materials. The same group took advantage of noncovalent dipolar interactions and developed self‐healable thermoplastic copolymers (Figure 2B).^[^ ^141^ ^]^ The self‐healing ability of the copolymers was attributed to the combination of dipolar interactions between C─F and C═ O moieties facilitating the self‐repair process. These pioneering studies highlight fundamental features of macromolecules, like the chain conformation and cooperative weak interactions, which can be explored for the design of structurally simple and functionally dynamic materials.

**Figure 3:**
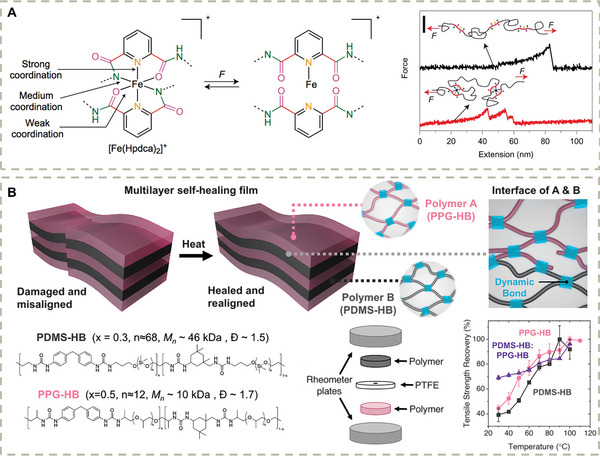
A) The design of highly stretchable and automous self‐healing material by incorporating coordination complexes with various bond strengths as cross–link. Reproduced with permission.^[^ ^142^ ^]^ Copyright 2016, Springer Nature Limited. B) The design of multilayer soft electronics with immiscible backbones and identical hydrogen bonding units. Reproduced with permission.^[^ ^143^ ^]^ Copyright 2023, American Association for the Advancement of Science.

A more general strategy to design self‐healing materials is based on the introduction of H‐bonds and metal‐ligand complexes, which have been discussed in Section 2.1. However, most of such materials exhibited relatively poor mechanical properties (either fragile or very soft). An important step towards simultaneously stretchable and self‐healable materials is achieved by Bao and coworkers by introducing an elegantly designed metal‐ligand complex (**Figure** **3**A),^[^ ^142^ ^]^ which combines strong metal‐ligand bonding and weak metal‐ligand bonding in a single system. The weaker metal‐ligand bonds can readily break and re‐form, while the stronger metal‐ligand interactions confine the metal ions allowing a rapid bond re‐formation. The cooperative effect of multiple metal‐ligand interactions enables the resulting materials to be highly stretchable and self‐healable, which exhibits potential applications in dielectric materials and actuators. Very recently, the same group achieved simultaneous autonomous realignment and healing in multilayer soft electronics by the combination of dynamic hydrogen‐bonding interactions and phase separation between different polymeric building blocks (Figure 3B).^[^ ^143^ ^]^ This concept facilitates the development of auto‐healable electronic devices.

**Figure 4:**
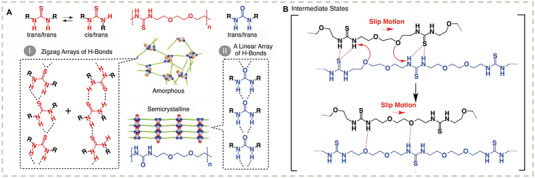
A) Schematic representations of the H‐bonding modes of thiourea and urea. B) Proposed mechanism of how the exchange of H‐bonded thiourea pairs is enhanced. Reproduced with permission.^[^ ^144^ ^]^ Copyright 2018, The American Association for the Advancement of Science.

Polymers relying on noncovalent interactions are relatively soft. Although some healable and robust polymer materials cross‐linked by dynamic covalent bonds have been investigated, high temperatures (normally higher than 120 °C) are needed for the reorganization of cross‐linked polymer networks.^[^ ^145^ ^]^ Aida and coworkers reported an amorphous poly(ether‐thiourea) polymer which can be cross‐linked by zigzag arrays of H‐bonds (**Figure** **4**).^[^ ^144^ ^]^ The dense but non‐crystalline H‐bond cross–linkers endowed the noncovalent network with mechanical robustness (Young's modulus up to GPa scale) as well as repairability at room temperature. A key discovery was that the spacer also significantly affected the dynamic chain mobility. Although the diffusion dynamics of the resulting polymer chains are low, the segmental motion such as the exchange of H‐bonded thiourea pairs leads to inter‐penetration of polymer chains while slip motion is favored by the polyether units, which is responsible for the rapid healing ability upon compression. This work showed for the first time that room‐temperature self‐healable materials can also be glass‐like with high mechanical robustness. The underlying mechanism based on this research provides a supramolecular model system that might be very useful to be further explored in the context of polymer physics.

**Figure 5:**
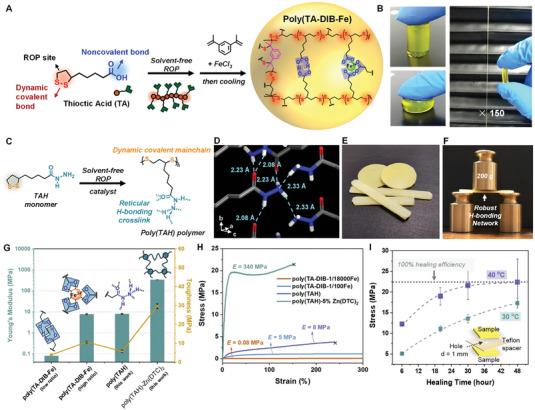
A) Molecular structure of TA and poly(TA‐DIB‐Fe) copolymer; B) Photographs showing the elastic and stretchability of the resulting poly(TA‐DIB‐Fe) materials. Reproduced with permission.^[^ ^119^ ^]^ Copyright 2018, The American Association for the Advancement of Science. C) Molecular structure of TAH and poly(TAH); D) Multiple H‐bonds observed in the single crystal structure of TAH monomers; E) Photograph of poly(TAH); F) Photograph showing the robustness of poly(TAH); G) Young's moduli comparison of poly(disulfide)s with different side chain cross–linkers; H) Tensile stress‐strain curves of different polymer samples; I) Self‐healing efficiency of poly(TAH)−5%Zn(DTC)_2_ polymers under ambient temperatures using a benchmark setup as shown in the inset scheme. Reproduced with permission.^[^ ^146^ ^]^ Copyright 2022, The American Association for the Advancement of Science.

Instead of engineering noncovalent interactions in polymer main chains, our group demonstrated the side chain engineering strategy to control the mechanical and dynamic properties of poly(disulfide) materials (**Figure** **5**). Using carboxylic monomer TA as the building blocks resulted in a soft (Young's modulus ≈80 kPa) and highly stretchable material that can be completely repaired in a few minutes owing to the cooperation of H‐bonds, metal‐ligand complexes, and dynamic covalent disulfide bonds (Figure 5A,B).^[^ ^119^ ^]^ Increasing the concentration of iron‐carboxylate complexes in the solvent‐free network led to the formation of ionic clusters and significant enhancement in stiffness without compromising stretchability.^[^ ^120^ ^]^ Recently, our group reported a sidechain H‐bond unit, acylhydrazine, that allowed the combination of mechanical robustness and dynamic behavior in a poly(disulfide) network (Figure 5C).^[^ ^146^ ^]^ The acylhydrazine group can be readily derived from carboxylic acids, while it shows distinctive H‐bond geometry compared with dimerized carboxylic acids. From the X‐ray single‐crystal structure of monomers (TAH, Figure 5D), every single acylhydrazine unit forms six H‐bonds with neighboring groups, resulting in the formation of a reticular H‐bond network with unique geometry, which is reminiscent of the ice crystal structure. Taking inspiration from this discovery, we further investigated the solvent‐free polymerization of TAH using a thermal melting procedure, obtaining a translucent yellow solid material. Mechanical tensile experiments showed that this noncovalent network exhibited high mechanical moduli (0.4 GPa) as well as high toughness and stretchability (Figure 5E–H), as a consequence of the noncovalent bonds acting as the sacrificial bonds to dissipate the external mechanical energy to avoid network breaking. Meanwhile, this robust noncovalent network still exhibited self‐healing ability under mild conditions (40 °C) due to the reversible exchange of acylhydrazine H‐bonds in the solvent‐free network (Figure 5I). This supramolecular network can also be used as high‐performance hot‐melt adhesives that exhibit state‐of‐the‐art adhesion performances for some substrates of industrial interests such as aluminum, glass, and steel.

**Figure 6:**
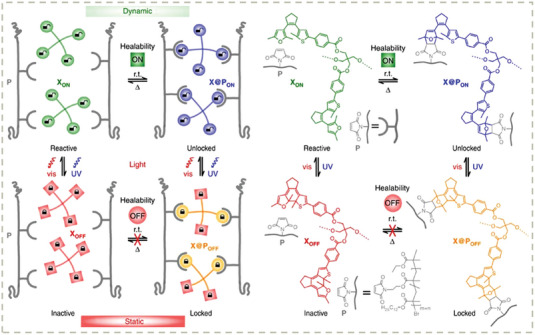
Schematic representation of light‐switchable cross–linker in its reactive and inactive state (left). The chemical structures of the tetrafuryl‐substituted diarylethene cross–linker (right). Adapeted with permission.^[^ ^147^ ^]^ Copyright 2016, Springer Nature Limited.

**Figure 7:**
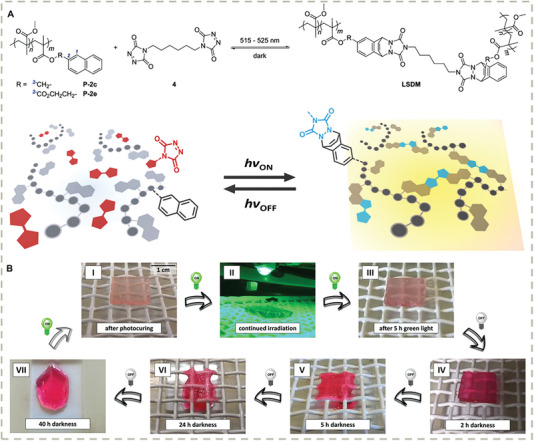
A) Synthesis of Light‐stabilized dynamic network. B) Macroscopic demonstration of light‐stabilized dynamic behavior. Reproduced with permission.^[^ ^148^ ^]^ Copyright 2019, American Chemical Society.

One of the most challenging issues to be addressed in self‐healing materials is how to balance the dynamicity and stability of materials. To enable self‐repairing property, the chain mobility must be allowed by dynamic cross–linkers that result in a fast chain exchange reaction at room temperature, which usually makes materials flow and creep. A feasible strategy to tackle this issue might be using light to switch the dynamicity and stability of the dynamic polymeric network under spatiotemporary control. Some recent photo‐dynamic covalent and noncovalent chemistries have been developed and used for smart self‐healing material design. An elegant design by Hecht, et al. demonstrated a furan‐containing diarylethene photoswitch used as photoswitchable dynamic cross–linkers for gel networks (**Figure** **6**).^[^ ^147^ ^]^ The key design is that the dynamic covalent Diels–Alder reactions can be reversibly activated or deactivated by light‐controlled ring opening and closing reactions of diarylethene units. As a consequence, the self‐healing ability of materials can be finely tuned by light, enabling localized activation and repair. Another important step towards light‐stabilized dynamic materials is the discovery of photo‐Diels‐Alder reaction of triazolinediones with naphthalenes (**Figure** **7**).^[^ ^148^ ^]^ The interesting feature of this system is the green‐light‐triggered formation of addition products, i.e., the cross–linking linkages, which autonomously decross–link in the dark. Thus, the topology of a polymeric material is a subtle compensation between photo‐induced cross–linking and thermal decross–linking. This conceptually novel dynamic covalent chemistry presents a structurally simple molecular tool for designing dissipative molecular systems and polymeric materials.

### 3.2 Processable Thermosets (Covalent Adaptable Networks, CANs)

Thermosets are commonly used materials due to their outstanding features of mechanical robustness and thermal/chemical stability. However, the presence of a permanently cross–linked polymer network makes them very difficult to be reprocessed or recycled to enhance the sustainability of the materials.^[^ ^81^, ^149^ ^]^ A challenge in the polymer industry is to develop polymeric materials that can be repeatedly mouldable and reprocessable like thermoplastics yet maintain the mechanical robustness of thermosets. Supramolecular materials exhibiting dynamic functions mostly bear low stiffness and poor thermal and chemical stability due to the intrinsic weakness and lability of noncovalent bonds. Thus, replacing the irreversible covalent cross–linking network in thermosets with dynamic covalent linkages offers an approach for constructing an adaptable cross–linked network simultaneously combining the robustness of covalent bonds with the reversibility of noncovalent bonds.

**Figure 8:**
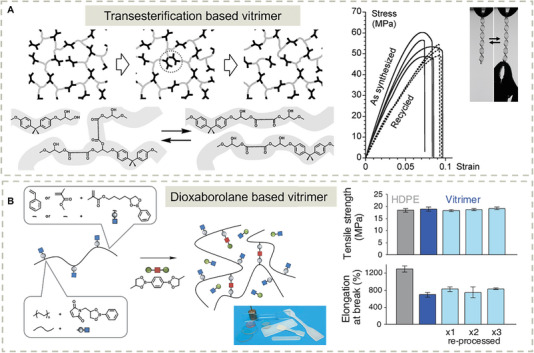
Vitrimers with a dynamic covalent cross–linking. A) Topological rearrangements via transesterification reactions preserve the network integrity in the hydroxy‐ester network. Reproduced with permission.^[^ ^150^ ^]^ Copyright 2011, The American Association for the Advancement of Science. B) Cross‐linking of functional polymers containing pendant dioxaborolane units by means of metathesis with a bis‐dioxaborolane. Reproduced with permission.^[^ ^151^ ^]^ Copyright 2017, The American Association for the Advancement of Science.

The concept of “vitrimer” was coined by Leibler in 2011,^[^ ^150^ ^]^ referring to malleable thermosets cross–linked by units with associable covalent linkages with adaptable topologies by thermally activated bond‐exchange reactions. Taking advantage of reversible transesterification reactions and inexpensive chemical building blocks, a vitrimer network was prepared, exhibiting silica‐like stiffness at room temperature, while becoming flowing and malleable at a temperature above 140 °C (**Figure** **8**A). The topology structure can be rearranged by the reversible ester exchange reactions without depolymerization and can maintain the total number of cross–links. The resulting polymers exhibit excellent malleability, reparability, and recyclability, and yet have solvent‐resistant properties making these new polymers a potential replacement for the composites of elastomers and thermosets in industry. The same group also introduced the metathesis reaction of dioxaborolanes into commercial polymers with carbon‐carbon backbones, such as poly(methyl methacrylate), polystyrene, and polyethylene by incorporating functional monomers during polymerization (Figure 8B).^[^ ^151^ ^]^ Benefiting from low‐cost commercial feedstocks, dioxaborolane‐based vitrimers bring possibilities for the large‐scale production of recyclable yet robust polymers. In recent years, several other dynamic covalent bond formations like siloxane exchange, and diketoenamines, have been applied in the design of vitrimer materials.^[^ ^152^, ^153^, ^154^, ^155^, ^156^ ^]^

**Figure 9:**
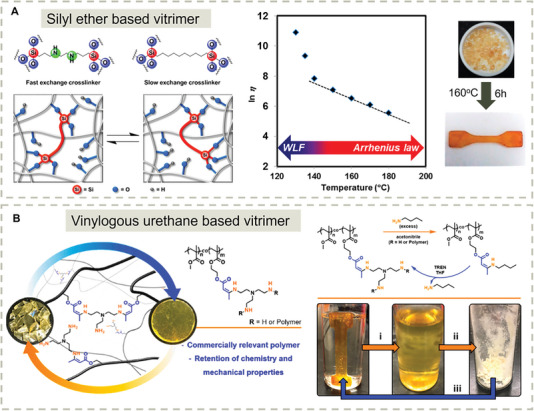
A) The design concept of tuning silyl ether exchange dynamics for vitrimer systems. Reprinted with permission.^[^ ^157^ ^]^ Copyright 2017, American Chemical Society. B) Amine exchange in vinylogous urethane vitrimer systems. Reproduced with permission.^[^ ^158^ ^]^ Copyright 2019, American Chemical Society.

The scope of applications of Si─O bonds in materials is extremely broad, ranging from soft silicon rubber to very hard inorganic glass. The dynamic exchange of silyl ethers in vitrimers was first developed by Guan and co‐workers (**Figure** **9**A).^[^ ^157^ ^]^ The key discovery was that the presence of a neighboring amino moiety could accelerate the dynamic exchange process of silyl ether by almost three orders of magnitude. This internal catalysis strategy made the cross–linked polystyrene vitrimers malleable by hot‐pressing at 160 °C for 6 h. Stress relaxation experiments also showed a transition from the Williams‐Landel‐Ferry (WLF) model to the Arrhenius model for the viscosity as a function of temperature due to the very special case that topology‐freezing temperature (*T*_v_, 47 °C) was lower than the glass transition temperature (*T*_g_, 125 °C) in this system. This discovery of new dynamic chemistry provides strategies for the design of dynamic organic silicon‐based materials.

Although catalysts can activate and accelerate the exchange of dynamic covalent cross–links to control viscoelastic properties, exogenous catalysts may degrade or leach from the polymer network over time. Hence, vitrimers without catalysts are preferred for practical applications. Sumerlin and co‐workers developed catalyst‐free vitrimers from commercially available feedstocks, like methyl methacrylate, (2‐acetoacetoxy)ethyl methacrylate, and tris(2‐aminoethyl)amine.^[^ ^158^ ^]^ The resulting catalyst‐free vinylogous urethane vitrimers maintain the high performance of vinylogous polymers meanwhile exhibiting healing properties allowing mechanical recycling. The vitrimers can also be decross–linked by adding excess monofunctional amine to achieve chemical upcycling (Figure 9B). These studies clearly demonstrated the versatility of the dynamic covalent toolbox for reconfigurating network architectures and material properties in a reversible manner.

### 3.3 Polymer‐to‐Monomer Recycling

To solve the problem of plastic pollution and achieve a closed‐loop sustainable circular economy, mechanical recycling by reprocessing or self‐repairing doesn't offer permanently sustainable materials because of the unavoidable fatigue in mechanical properties and the limited upcycling space to increase the economic effectiveness of the recycled materials.^[^ ^159^ ^]^ Meanwhile, sorting out commodity post‐consumer waste [e.g., polyethylene and polypropylene ] is still a major challenge for mechanical recycling.^[^ ^160^, ^161^, ^162^ ^]^ For numerous common plastic materials, external additives, such as plasticizers, heat stabilizers, metal deactivators, dyes, and flame retardants, not only inhibit the effective mechanical recycling of the end products but also result in loss of mechanical performance during mechanical recycling.^[^ ^163^ ^]^

On the other hand, chemical recycling can provide a solution for achieving virgin‐quality monomer recovery from the depolymerization of plastic waste.^[^ ^160^, ^164^ ^]^ Although the concept of depolymerization of synthetic polymers has been proved for supramolecular polymers, the bottleneck towards the next‐generation alternative polymers rests on the question of how to achieve chemically recyclable synthetic polymers with mechanical performances that are comparable with common commercial plastics.^[^ ^149^, ^160^ ^]^ Therefore, it remains a major challenge to develop a synthetic polymer that behaves like commercial plastics but simultaneously can be chemically recycled into virgin‐quality monomers in a cost‐effective manner.

**Figure 10:**
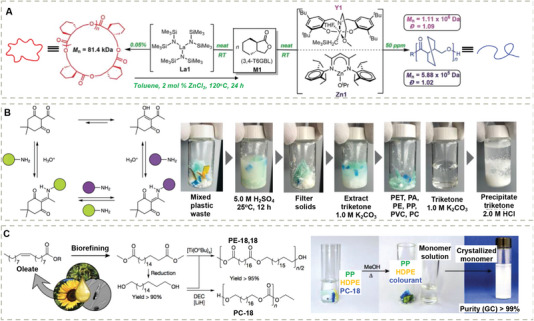
A) Chemically recyclable polymers from controlled ROP of cyclic esters. The topology can be tuned by catalysts. Reproduced with permission.^[^ ^165^ ^]^ Copyright 2018, The American Association for the Advancement of Science. B) Reversible, dynamic covalent diketoenamine bonds and photographs of the depolymerization and recycling of poly(diketonenamine) in strong acid aqueous solution from a plastic waste mixture. Reproduced with permission.^[^ ^166^ ^]^ Copyright 2019, Springer Nature Limited. C) A high‐performance semi‐crystalline thermoplastic derived from biobased oleate can be readily recycled by depolymerization in methanol. Reproduced with permission.^[^ ^169^ ^]^ Copyright 2021, Springer Nature Limited.

Chen and co‐workers reported a synthetic polyester system based on a γ‐butyrolactone (**Figure** **10**A).^[^ ^165^ ^]^ Due to the additional trans‐ring fusion, the hard‐to‐polymerize γ‐butyrolactone unit could be solvent‐free polymerized at room temperature. The resulting polymers show high mechanical performance with breaking tensile stress at (*σ*_b_) = 55 MPa, high molecular weight (*M*_n_ = 145 kg mol^−1^), and enhanced thermostability [decomposition temperature (*T*_d_) = 340 °C], yet could also be chemically recycled to monomers by thermolysis at ≥ 300 °C for 1 h or by chemolysis with catalyst (ZnCl_2_) at 120 °C. This pioneering study demonstrated an efficient chemically recyclable synthetic polymer by engineering the thermodynamic equilibrium of ring‐opening polymerization by exploring the ring strain of monomers, opening up a new avenue to the rational design of chemically recyclable covalent polymers.

Although high temperatures and catalysis could accelerate the depolymerization process, the high energy cost compromises its sustainability while the presence of catalysts, to some extent, might affect the durability of the recycled materials. Dynamic covalent chemistry offers a solution enabling intrinsic dynamicity based on the reversible linkage in polymeric networks. Helms and co‐workers reported the closed‐loop chemical recycling of covalently cross–linked networks by dynamic covalent diketoenamine bonds (Figure 10B).^[^ ^166^ ^]^ The resulting poly(diketonenamine) materials could be decross–linked at room temperature in an aqueous solution of strong acids, such as H_2_SO_4_ or HCl, and the two monomers, i.e., the amines and the ketones, can be separated through a closed‐loop procedure. This proof of concept shows how dynamic covalent chemistry can be used for the design of a chemically recyclable cross–linked network. Several follow‐up studies have been performed by the same group to give more mechanistic insights and environmental assessment of this new technology.^[^ ^167^, ^168^ ^]^

Polyethylene based plastics are among the most used materials in daily life, such as plastic bags, films, tubes, etc. Finding a greener substitute for it is very difficult because of the very low production cost of polyethylene and well‐established methodologies on how to tune their mechanical properties by polymer crystallization. An elegant example recently presented by Mecking and co‐workers took advantage of the dynamic covalent nature of ester bonds, enabling the closed‐loop chemical recycling of polyethylene‐like materials by introducing low densities of ester bonds as cleavable moieties in the polyethylene chain. Meanwhile, the starting materials can be readily obtained by biorefining from biobased feedstocks (Figure 10C).^[^ ^169^ ^]^ The key discovery is that introducing periodically spaced ester bonds between long alkyl chains not only preserves polyethylene‐like crystallization that enables high mechanical performances but also endows base‐catalyzed depolymerization by hydrolysis of the ester bonds. Depolymerized monomers can be easily separated from methanol by simple crystallization‐induced precipitation with high yields and purity.

**Figure 11:**
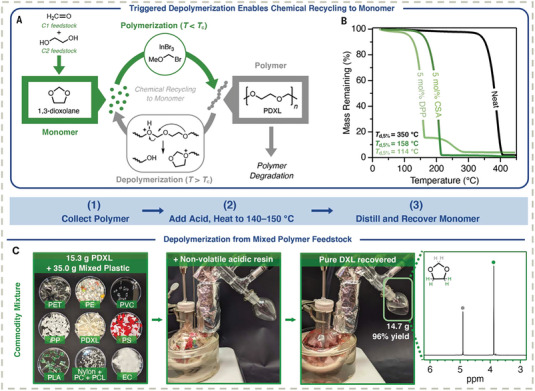
A) Acid‐catalyzed reversible polymerization and depolymerization of poly(1,3‐dioxolane) (PDXL). Reprinted with permission.^[^ ^93^ ^]^ Copyright 2021, The American Association for the Advancement of Science. B) Thermal stability of poly(1,3‐dioxolane) before and after adding 5 mol% camphorsulfonic acid or diphenylphosphoric acid. Reprinted with permission.^[^ ^93^ ^]^ Copyright 2021, The American Association for the Advancement of Science. C) Recycling experiment setup enabling nearly quantitative isolated yield and high purity from the mixture of poly(1,3‐dioxolane) and some commodity plastics feedstocks. Reproduced with permission.^[^ ^93^ ^]^ Copyright 2021, The American Association for the Advancement of Science.

Polyacetals are another potential candidate for chemical recycling to monomers, but their molecular weight is difficult to control by the traditional cationic ring‐opening polymerization (ROP) method of 1,3‐dioxolane.^[^ ^170^ ^]^ Coates and co‐workers proposed a reversible‐deactivation ROP method, where the initiator (chloromethyl methyl ether), catalysts (Lewis acid InBr_3_), and proton trap (2,6‐di‐tert‐butyl pyridine) were added to the system to control the molecular weight.^[^ ^93^ ^]^ The resulting poly(1,3‐dioxolane) exhibited comparable mechanical properties to some commodity polyolefins. The depolymerization of poly(1,3‐dioxolane) could be achieved by adding strong acids, such as camphorsulfonic acid or diphenylphosphoric acid, and even in a commodity plastic waste mixture an isolated yield of up to 96% was achieved (**Figure** **11**).

**Figure 12:**
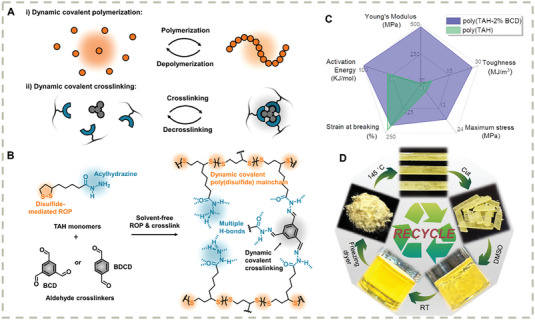
A) Conceptual illustration of the orthogonal dynamic covalent chemistry (disulfide, and hydrazone) for chemical recycling of cross–linked network; B) Molecular structures of monomers and polymers; C) Mechanical performances of covalently cross–linked poly(TAH‐2%BCD) and noncovalent network poly(TAH); D) Chemical recycling procedures of the materials. Reproduced with permission.^[^ ^171^ ^]^ Copyright 2022, Wiley‐VCH GmbH.

Recently our group proposed orthogonal dynamic covalent chemistry for chemical recycling of a covalently cross–linked network (**Figure** **12**A).^[^ ^171^ ^]^ Using acylhydrazine‐modified monomer (TAH) and aldehydes (1,3,5‐benzenetricarbaldehyde (BCD) and benzene‐1,4‐dicarboxaldehyde (BDCD)) as the starting materials, a cross–linked network can be prepared in one pot by solvent‐free ROP (Figure 12B). Introducing minimal amounts (2%) of BCD cross–linkers can significantly improve the mechanical performance and durability of the network based on acylhydrazone formation (Figure 12C). The cross–linked material showed stability under most solvents except dimethyl sulfoxide (DMSO), in which the material depolymerizes into a yellow monomeric solution (Figure 12D) following an acylhydrazine‐catalyzed ring‐closing depolymerization mechanism. Virgin‐quality monomers can be recovered by freeze‐drying from the DMSO solution. This is a proof of concept that orthogonalizing two types of dynamic covalent chemistry, i.e., disulfide and hydrazone, in a single system can be an effective dynamic chemistry strategy to design chemically recyclable cross–linked materials.

### 3.4 Chemical Upcycling of Commodity Plastic Wastes

Instead of designing new polymers and materials, a more realistic manner might be developing chemical upcycling methodologies to transform commodity plastic wastes into value‐added chemical products. This route doesn't close the recycling loop, while it might be able to tackle the current plastic problems in a cost‐effective way because it directly uses plastic wastes from modern industry as feedstocks. Many catalytic strategies have been developed to degrade plastics into fuels, surfactants, and monomers. In this section, we would also like to highlight some very recent progress in enabling upcycling of commodity plastic wastes using dynamic chemistry tools.

**Figure 13:**
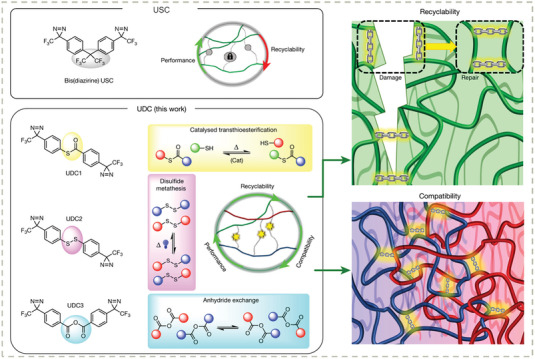
Design of universal dynamic cross–linkers and their molecular and macromolecular features. The thermosets are reprocessable and compatible with immiscible mixed plastics via dynamic bond exchange. Reproduced with permission.^[^ ^172^ ^]^ Copyright 2023, Springer Nature Limited.

Chen and Rovis, et al. reported a series of dynamic covalent cross–linkers for the compatible upcycling of several types of mixed plastics that are physically immiscible (**Figure** **13**).^[^ ^172^ ^]^ The key design strategy is combining dynamic covalent chemistry (e.g., disulfide bonds, transthioesterification, and anhydride exchange) with the photo‐triggered C─H activation cross–linking strategy of bis(diazirine) units pioneered by Wulff, et al.^[^ ^173^ ^]^ This molecular “glue” interfaces the nonpolar and polar backbones of different types of mixed plastics to enable molecularly repaired interfaces, i.e., compatibility. As a result, polyesters and polyolefins can be simultaneously processed into graft multiblock copolymers that exhibit intrinsic reprocessability and enhanced mechanical strength and creep resistance compared with original plastics. This strategy should bear conceptual generality to any kind of polymers containing C─H bonds. The system might be a bit cost‐ineffective since current building blocks are still synthetically tedious and cost‐ineffective compared with the millions of tons of plastic waste. However, the conceptual significance is clear and would be extremely inspiring for the community of plastic recycling and dynamic chemistry.

**Figure 14:**
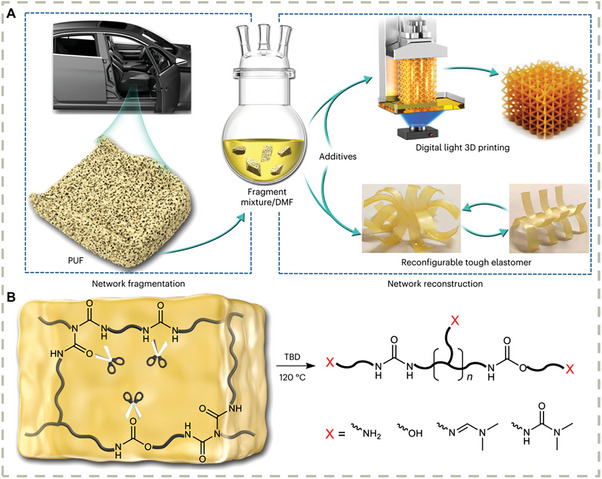
A)The chemical fragmentation and upcycling of commodity thermoset polyurethane foams. B) Chemical fragmentation of polyurethane into dissolvable polymer fragments. Reproduced with permission.^[^ ^174^ ^]^ Copyright 2023, Springer Nature Limited.

Another very recent example from Xie, et al. reminds that many commodity plastics that are thought of as “inert” materials can also be intrinsic to dynamic covalent nature (**Figure** **14**).^[^ ^174^ ^]^ They discovered that polyurethane thermosets can be dissolvable in dimethylformamide (DMF) in the presence of 1,5,7‐triazabicyclo[4.4.0]dec‐5‐ene (TBD) as an organic base catalyst and chemically upcycled into soluble oligomers terminated with amine, ol, secondary urea, and amidine moieties, which can be further functionalized and cured into high‐performance thermosetting materials suitable for 3D‐photo‐printing production. The concept in this work is to explore the “intrinsic dynamicity” in traditional polymeric materials instead of reinventing a new material. The mild conditions, excellent material properties, and ability in material manufacture jointly make this dynamic chemistry represent an important step towards the cost‐effective upcycling of commodity plastics.

## 4 Summary and Outlook

By discussing the design principles and highlighting recent advances toward the development of dynamic chemistry for sustainable materials, it is evident that this strategy shows great promise for building sustainable plastic materials of the future. These examples represent a starting point for how to use dynamic covalent and noncovalent toolboxes to enable sustainability from the fundamental design of molecular structures to the macroscopic functions of materials and their recycling capability. Facing the challenge of fast recycling, it is to be expected that the number of dynamic polymeric materials will exponentially increase in the next decades. Therefore, we would like to present a brief outlook for future challenges and opportunities in this field.

Several examples have shown that combining two or more types of dynamic chemistries in a single system could enable unique materials that cannot be realized solely by a single dynamic system. This combination can be orthogonal, synergistic, or cooperative. In most cases, orthogonal systems have been developed so far. Synergistic noncovalent and dynamic covalent materials are getting more attraction in current research, indicating there is still a large chemical space to be explored. Key principles should be studied in how to make these two types of dynamic chemistry operate to achieve the proper balance in stability and dynamic behavior. Thermodynamic analysis should be performed to give a quantitative insight into the subtle interplay between the two coupled equilibrium systems, and how this effect makes a substantial change in the macroscopic dynamic functions at the material level.

Furthermore, environmental assessment should be introduced as a benchmark for evaluating the processing impact on the environment and the economic efficiency of the preparation and recycling routes of a material. This is crucial because these endeavors are not solely aimed toward fundamental studies, but also at solving real problems associated with current plastics. Conducting such programs also requires a more standard description of the experimental methods and parameters, and assessing the scalability and practicality using the proposed future green chemistry principles.^[^ ^11^ ^]^

It is evident that the development of dynamic chemistry has brought many opportunities for the design of advanced sustainable materials aiming for future applications. Besides the emerging examples that we have summarized in Section 3, we foresee that the next step of this field will be the integration of multi‐dynamic functions in a single material, i.e., one material combining the advantages of repairing, reprocessing, and recycling. This aim is realistic because the fundamentals of dynamic covalent and noncovalent chemistries are developing remarkably quickly and the fields are merging rapidly. We have seen a few examples such as the poly(thioctic/lipoic acid) system featuring many attractive functions in a single material.^[^ ^120^, ^122^, ^123^, ^125^, ^127^, ^146^ ^]^ In the future, it can be envisioned that more classes of “dual dynamers” or even “multi‐dynamers” will be developed and they are likely going to play a key role in material design and functional applications.

Meanwhile, we are also facing many challenges to achieve practically applicable dynamic materials as suitable candidates to replace the use of traditional plastics. One of the major challenges is also the control of cost. Traditional plastics have been invented and optimized for a century, which has resulted in an extremely mature industry allowing large scalability and low production cost. Our lab‐invented materials have at least, been made comparable with this “previously thought most successful chemical product” both in quality and cost as well as combining with dynamic functions enabling sustainability and the urgency of this task is evident. To call for more input and contributions from diverse communities, a few challenges in terms of dynamic chemistry and materials are summarized below:

- How to overcome the inherent trade‐off between mechanical robustness and dynamic properties in material design?
- How to design intrinsically dynamic materials that can be readily repaired and recycled under mild conditions?
- How to achieve “green in source” by using biomass, instead of petroleum‐based compounds to produce dynamic sustainable materials?
- What is the interface between noncovalent and dynamic covalent chemistry? How to combine the two dynamic chemistries to generate synergistic dynamic materials?
- What kinds of functional applications can be exploited by using dynamic polymers?

One should also realize that we are still at the very early stage of proof of concept on how to make self‐healing materials and how to recycle polymers. Current technology is far away from the economically efficient replacement of traditional plastics using the potential “green” polymers presented in this review. It remains challenging, how to avoid the use of expensive catalysts, how to minimize the synthetic cost, how to maximize the materials functions, and how to recycle them sustainably while retaining properties and durability. To this end, we might get some inspiration from living systems: Our body is a circular recycling system so we don't need too many supplements for daily use. Our enzymes are efficient in digesting (depolymerizing) long peptides into amino acid monomers by breaking the robust amide bonds, which looks a formidable challenge considering the state of current dynamic chemistry. But we are confident that, with the multidisciplinary development of modern chemistry and fully integrated dynamic features a greener and smarter material future will come soon.

## Conflict of Interest

The authors declare no conflict of interest.
